# Investigation of Laser Welding of Ti Alloys for Cognitive Process Parameters Selection

**DOI:** 10.3390/ma11040632

**Published:** 2018-04-20

**Authors:** Fabrizia Caiazzo, Alessandra Caggiano

**Affiliations:** 1 Department of Industrial Engineering, University of Salerno, 84084 Fisciano (SA), Italy; f.caiazzo@unisa.it; 2 Department of Industrial Engineering, University of Naples Federico II, 80125 Naples, Italy; 3 Fraunhofer Joint Laboratory of Excellence on Advanced Production Technology (Fh-J_LEAPT UniNaples), 80125 Naples, Italy

**Keywords:** laser welding, titanium alloys, artificial neural networks

## Abstract

Laser welding of titanium alloys is attracting increasing interest as an alternative to traditional joining techniques for industrial applications, with particular reference to the aerospace sector, where welded assemblies allow for the reduction of the buy-to-fly ratio, compared to other traditional mechanical joining techniques. In this research work, an investigation on laser welding of Ti–6Al–4V alloy plates is carried out through an experimental testing campaign, under different process conditions, in order to perform a characterization of the produced weld bead geometry, with the final aim of developing a cognitive methodology able to support decision-making about the selection of the suitable laser welding process parameters. The methodology is based on the employment of artificial neural networks able to identify correlations between the laser welding process parameters, with particular reference to the laser power, welding speed and defocusing distance, and the weld bead geometric features, on the basis of the collected experimental data.

## 1. Introduction

Titanium alloys are widely employed in the aerospace sector, due to high strength in combination with low density and good tensile properties; moreover, resistance to corrosion allows for applications in chemically aggressive conditions. In particular, the Ti–6Al–4V alloy accounts for more than half of all titanium tonnage in the world, and no other titanium alloy appears to threaten its dominant position. The use of the Ti–6Al–4V alloy is widespread in the aerospace sector, for turbine disks, compressor blades, airframe and space capsule structural components, rings for jet engines, pressure vessels, rocket engine cases, helicopter rotor hubs, fasteners, and engine exhausts.

Compared to fully-machined products, the employment of assembled products allows a reduction in waste material, and leads to shorter lead times and a lower buy-to-fly ratio (i.e., the ratio between the weight of the raw material used to manufacture a part and the actual weight of the finished part) [1]. As regards the joining techniques, recent improvements at the design stage of aerospace components are aimed at removing any mechanical fastening, such as screwing and riveting, to introduce welded assemblies and to avoid any increase of part thickness.

Laser welding of titanium alloys is therefore attracting increasing interest as an alternative to traditional joining techniques for industrial applications [2]. The particular advantages of this process, compared to a TIG (Tungsten Inert Gas) or plasma arc welding, include an increased depth of penetration, a decrease of possible welding defects, and reduction of the melted and heat-affected zone [3], thus entailing an enhancement of the mechanical performance of the welded structures [4]. Another innovative method of welding with a highly concentrated energy source is electron beam welding [5], but this technique presents the disadvantage that a vacuum is needed to perform the process; in addition, the electron beam welding process involves X-rays emissions.

In comparison with traditional technologies, tight laser beams have been proved to be effective in reducing both angular distortion and longitudinal bending on thin sheets. Furthermore, a reduced mean grain size in the fusion zone is achieved; the overall mechanical quality is hence improved, considering that the grain growth is deemed to be one of the reasons for the reduction of tensile strength upon welding.

For these reasons, a number of scientific studies are available in the literature dealing with laser beam welding of titanium alloys [6,7], which are widely used in aerospace thanks to their high strength in combination with low density and good tensile properties; medical and surgical devices are even produced using these alloys, thanks to high biocompatibility [8].

However, the relationship between the operational process parameters and the resulting weld bead properties is complex, and efforts have been made to develop various mathematical tools for the prediction and optimization of weld bead characteristics.

In this research work, an investigation of laser welding of Ti–6Al–4V alloy plates is carried out through an experimental testing campaign under different process conditions, in order to perform a characterization of the produced weld bead geometry, with the final aim of developing a cognitive methodology able to support decision making regarding the selection of the suitable laser welding process parameters, in order to obtain a weld bead with defined geometry. The cognitive methodology is based on the employment of artificial neural networks able to identify correlations between the laser welding process parameters, with particular reference to the laser power, welding speed and defocusing distance, and weld bead geometric features, on the basis of a training performed by feeding the network with the collected experimental data.

Artificial neural networks (ANN) are able to provide non-linear mapping between a set of input variables and a set of output variables. In many manufacturing process applications, ANN have been employed in a forward manner, i.e., to calculate the response in terms of output quality parameters, based on the values of specified input process parameters. This is the case, for instance, with the work carried out in [9], where an artificial neural network was designed in order to calculate penetration-to-fuse-widths and penetration-to-haz-widths for different laser powers, welding speeds, and focal positions. In [10], an ANN-optimization hybrid model was proposed for prediction and optimization of penetration for different welding parameters in CO_2_ LASER-MIG welding. Four controllable welding parameters were taken as input, namely power, focal distance from the work piece surface, torch angle, and distance between the laser and welding torches. The depth of weld penetration was provided as ANN output, to assess the effect of input parameters on this response. However, ANN can be employed to realize both forward and reverse mapping. In [11], ANN were applied to illustrate the correlation between the input and output responses in friction welding of Incoloy 800 H. Four process parameters (heating pressure, heating time, upsetting pressure, and upsetting time) and three responses (tensile strength, micro-hardness, and burn off length) were considered. The mapping of the process in the reverse direction, i.e., feeding the desired responses as inputs to the model and obtaining, as outputs, the process parameters required to achieve the desired set of responses. This is a valuable support for decision making about the selection of the suitable process parameters, and represents a relevant issue for process automation [10]. According to the reverse mapping approach, ANN were employed in the present research work, with the aim of estimating the laser welding process parameters (laser power, welding speed, and defocusing distance) required to obtain a weld bead with defined geometrical features (bead crown width, root width, heat affected zone width on the top surface, heat affected zone width on the bottom surface, and area of the fused zone). Accordingly, the measured geometric features of the weld bead obtained from the experimental testing campaign were employed as input to train the ANN, while the estimated process parameters represented the ANN output.

## 2. Materials and Methods

A Ti–6Al–4V alloy (Table 1) in 3 mm thick sheets was considered for producing welding beads in a butt configuration. Samples had a length of 100 mm (along the welding direction) and a width of 50 mm. In the field of laser welding, and considering aerospace applications, the preparation of the joints to ensure proper matching of the surfaces and remove foreign particles that could contaminate the melt pool is relevant. Therefore, top surfaces were milled, and any burrs were removed with abrasive paper. A diode-pumped disk-laser source, characterized by a focal beam diameter of 0.3 mm, was used to produce the welding samples in a continuous wave emission. This method is advantageous, because only weak thermal lensing effects are in place when using a thin disk source; better beam quality is obtained, because the divergence and the diameter variation along the propagation axis is restrained. In particular, a beam parameter product of 8 mm × mrad is achieved for the welding system in use, with a resulting Rayleigh range of 2.81 mm. The technical data of the welding system are listed in Table 2.

Since titanium alloys are prone to oxidation when in a fused state, it has been proven that gas shielding is crucial for bead protection, in order to obtain sound joints. Therefore, a specific device to protect the joint must be considered. As discoloration of the bead is evidence of oxidation taking place during welding, visual inspections are generally conducted to preliminarily assess the effectiveness of bead shielding. With respect to the assisting gas, which is additionally required to the purpose of metal plume removal, it has been shown that deeper penetration is achieved when considering helium instead of argon, thanks to the higher ionization potential of the former.

**Figure 1:**
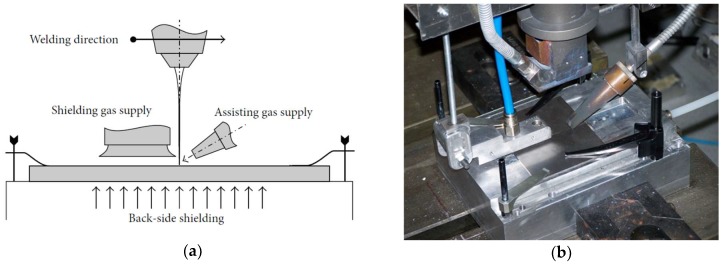
(**a**) Laser welding system scheme and (**b**) gas shielding system picture.

As bead shielding is crucial to obtain sound joints when processing titanium alloys, a specific device to protect the joint must be considered. The device employed for the experimental tests (Figure 1) consists of a side diffuser and a grooved box for top- and back-side shielding, respectively [12]. Argon was considered for both top-side and back-side shielding; an air cross-jet prevented metal drops from reaching the optics. The gas flow rate, welding direction, nozzle angle, and diffuser position were chosen based on trial experiments. Argon, at a flow rate of 10 L/min, was selected for the top side, with a flow rate of 50 L/min chosen for back-side shielding, while helium was preferred as the assisting gas for plume removal via a leading nozzle, with a flow rate of 20 L/min.

A proper choice of the governing parameters was based on preliminary experiments. Laser power, *p*, and welding speed, *s*, were taken into consideration in the study, as they are primary factors which determine the rate of energy input to the work piece. In addition, successful laser welding requires optimization of other parameters, such as the size and location of the focal spot.

The defocusing distance, *f*, which is the distance of the focal point with respect to the top surface, was included in the experimental plan, as changes in focus position directly affect the weld width and depth. In particular, defocusing is intended to be positive or negative when the focal point is located above or beneath the top surface, respectively. Negative defocusing, i.e., with the beam focal point beneath the metal surface, was considered because it has been associated with a reduction of the grain size; the growth in grain size would otherwise produce a decrease in the tensile strength of the welded structures.

Factorial experiments were planned. A three-level (levels −1, 0, +1) experimental plan, with laser power (*p*), welding speed (*s*), and defocusing distance (*f*) as governing factors was arranged; the factor levels for each parameter are listed in Table 3.

**Figure 2:**
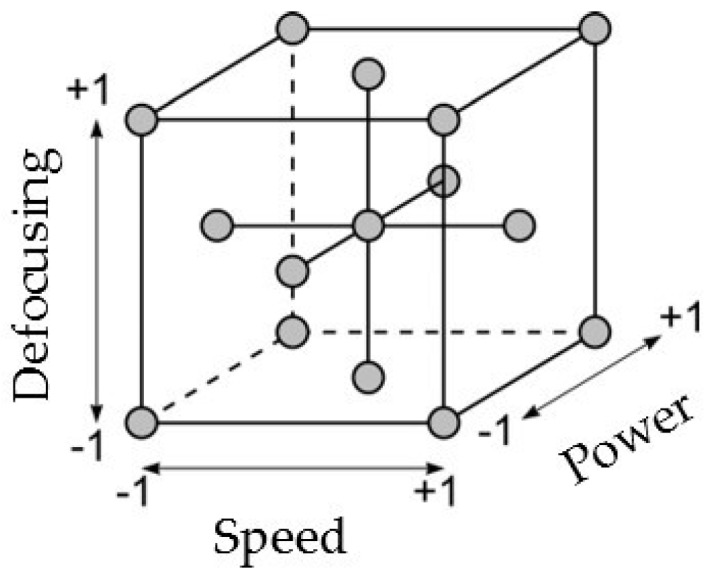
Face-centered central composite design scheme.

A fractional design was preferred, with the aim of reducing the number of experimental tests. A central composite design was planned; a face-centered scheme was chosen, in order to explore the areas within the ranges previously found in preliminary trials. The tests to be performed are placed on a cubic lattice, according to Figure 2. Three runs were planned for each process condition, in order to check the statistical significance of measurements. A random test procedure was arranged, both to allocate the plates and to produce the specimens, so that the observations are independent random variables, with the aim to reduce systematic experimental variation.

In order to investigate the weld bead geometry produced by the laser welding experimental tests under different process parameters, the butt samples were cross-cut perpendicularly to the welding direction, and then polished to a mirror finish with SiC paper and grinding diamond paste on polishing cloths. Chemical etching was performed using a solution of hydrofluoric acid (48%, 10 mL), nitric acid (65%, 15 mL), and water (75 mL) at room temperature, in order to highlight the bead boundaries in the cross-section.

**Figure 3:**
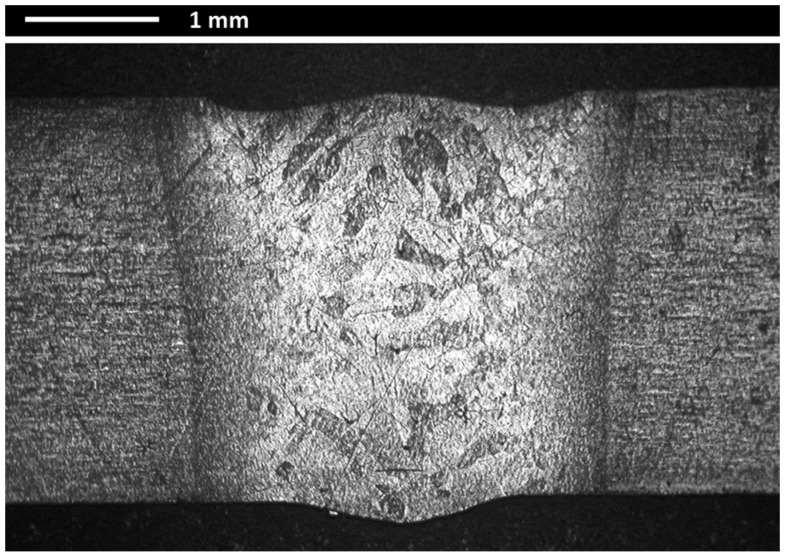
Cross-section micrograph of the specimen corresponding to the center point of the plan.

An overview of the transverse cross-section of the specimen in the center experimental testing condition (*p* = 1750 W, *s* = 20 mm/s, *f* = −1.5 mm) is shown in Figure 3.

**Figure 4:**
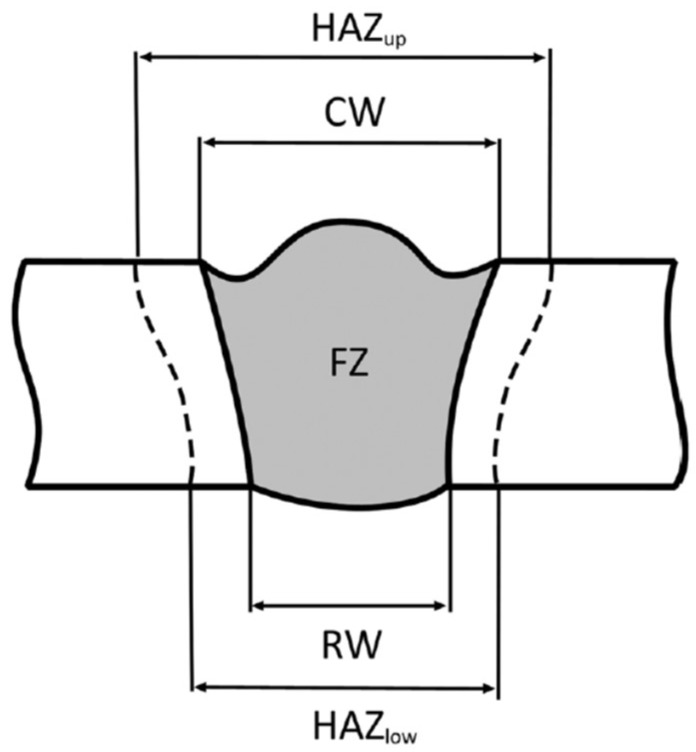
Geometric features employed for weld bead characterization.

To characterize the bead shape, the following geometric responses, shown in Figure 4, were measured: the bead crown width (CW), the root width (RW), the width of the heat-affected zone on the upper surface (HAZup), the width of the heat-affected zone on the lower surface (HAZlow), and the area of the fused zone (FZ). These geometric features were measured via lineal analysis using a dedicated imaging software. For each welding condition, three reference lines in three different positions in the same cross-section were considered.

Over all the planned experimental testing conditions shown in Table 3 and Figure 2, three conditions were not able to successfully perform the welding process, due to low penetration depth. These conditions were *p* = 1500 W, *s* = 15 mm/s, and *f* = −3 mm; *p* = 1500 W, *s* = 25 mm/s, and *f* = −3 mm; and *p* = 1500 W, *s* = 25 mm/s, and *f* = 0 mm; all characterised by the lowest power value among the three levels considered in the design of experiments. All the other experimental testing conditions provided bead welds with complete penetration in the 3-mm-thick Ti–6Al–4V plates. The weld bead geometric features measured via micrographic analysis on these samples are reported in Table 4, together with the process parameters employed during the experimental tests.

## 3. Cognitive Process Parameter Selection Based on Artificial Neural Networks

The experimental data acquired through the Ti–6Al–4V alloy laser welding tests were used to develop a cognitive methodology to support decision making on the selection of the suitable process parameters, to achieve a weld bead with the desired geometrical features. The proposed cognitive methodology is based on the employment of artificial neural networks (ANN) for the identification of correlations between the laser welding process parameters (laser power *p*, welding speed *s*, and defocusing distance *f*) and the output weld bead geometrical parameters (CW, RW, HAZup, HAZlow, and FZ).

Artificial neural networks were selected, as they offer a very powerful and general framework for representing nonlinear mappings between a set of input and output variables. Compared to other non-linear mapping methods, ANN represent a more flexible and self-adjusting approach that can accommodate various types of nonlinear behavior, representing a broad class of nonlinear approximations and mappings, and conveniently deal with problematic dimensionality issues that arise when the dimensionality of the input space is high [13].

The measured geometric features obtained from the experimental testing campaign were employed to train different ANN architectures for the estimation of the appropriate process parameters. In particular, the five geometric features (CW, RW, HAZup, HAZlow, and FZ) measured on the weld bead produced by each experimental laser welding test were combined to build feature pattern vectors (FPV) that were used as input for ANN learning [14]. Only the data from the experimental tests for which complete penetration depth was achieved, listed in Table 4, were used for the ANN training, obtaining a training set of *m* = 36 FPV (12 different combinations of laser welding process parameters, each repeated three times for statistical significance).

Each training vector was a five-feature FPV, composed as follows:

FPV_j_ = [*CW_j_*, *RW_j_*, *HAZup_j_*, *HAZup_j_*, *FZ_j_*]  *j* = 1, …, *m* (1)

Each five-feature FPV_i_ was matched to the related process parameter combination (*p_j_*, *v_j_*, *s_j_*), which represents the ANN output, to carry out supervised ANN learning.

Output_j_ = [*p_j_*, *v_j_*, *s_j_*]  *j =* 1, …, *m* (2)

The selected ANN type was a three-layer, cascade-forward, backpropagation ANN architecture, using the Levenberg–Marquardt algorithm as an ANN training function, and the tan-sigmoid as the transfer function. The number of epochs was established equal to 1000, and the minimum performance gradient was set to 1 × 10^−7^. Multiple ANN configurations characterized by different numbers of hidden layer nodes were employed, in order to compare each one’s performance and select the best-performing ANN configuration. The number of input layer nodes was always equal to 5, corresponding to the dimensionality of the input FPV, and the number of output layer nodes was set to 3, i.e., equal to the number of output process parameters to be estimated. The different ANN configurations were characterised by diverse numbers of hidden layer nodes, ranging from 1× to 3× the number of input layer nodes, i.e., from 5 to 15 nodes.

The training set of *m* = 36 FPV was employed to train the ANN, i.e., to calculate the gradient and update the ANN weights and biases [13,15]. To evaluate the performance of the trained ANN, a testing set was built to employ the average values of the measured geometric features (CW, RW, HAZup, HAZlow, and FZ) of the weld bead, which were computed over the three repetitions of each experimental testing condition. The testing set was therefore composed of *n* = 12 FPV, equal to the number of different experimental testing conditions listed in Table 4, where each FPV consisted of a combination of the average values of the measured geometric features. To evaluate the global pattern recognition performance of an ANN configuration, the *n* pattern recognition rates achieved over the entire testing set were aggregated.

The pattern recognition performance of each ANN configuration was evaluated for each output parameter (i.e., laser power *p*, welding speed *s*, and defocusing distance *f*), in terms of root mean square error (RMSE) between the ANN-predicted parameter values (*p_ANN_*) and the target parameter values (*p_T_*).

(3) $$\mathrm{RMSE}\;=\;\sqrt{\frac{\sum_{t\;=\;1}^{n}{\left(p_{ANN}-p_{T}\right)}^{2}}{n}}$$

The performance rates obtained by the different ANN configurations, with different numbers of hidden layer nodes, allowed the selection of the optimal ANN configuration, which is the one with 14 hidden layer nodes. The aggregated RMSE values obtained by this configuration over the *n* = 12 testing cases were equal to 117.10 W for laser power, 1.77 mm/s for welding speed, and 0.16 mm for defocusing distance. These values can be considered absolutely satisfactory, taking into account the following aspects: the minimum distance between the three power levels was of 250 W, hence more than double compared to the ANN error on this parameter; the minimum distance between the three welding speed levels was of 5 mm/s, i.e., more than double that of the ANN error on this parameter; the minimum distance between the three defocusing distance levels was of 1.5 mm/s, i.e., more than double that of the ANN error on this parameter.

**Figure 5:**
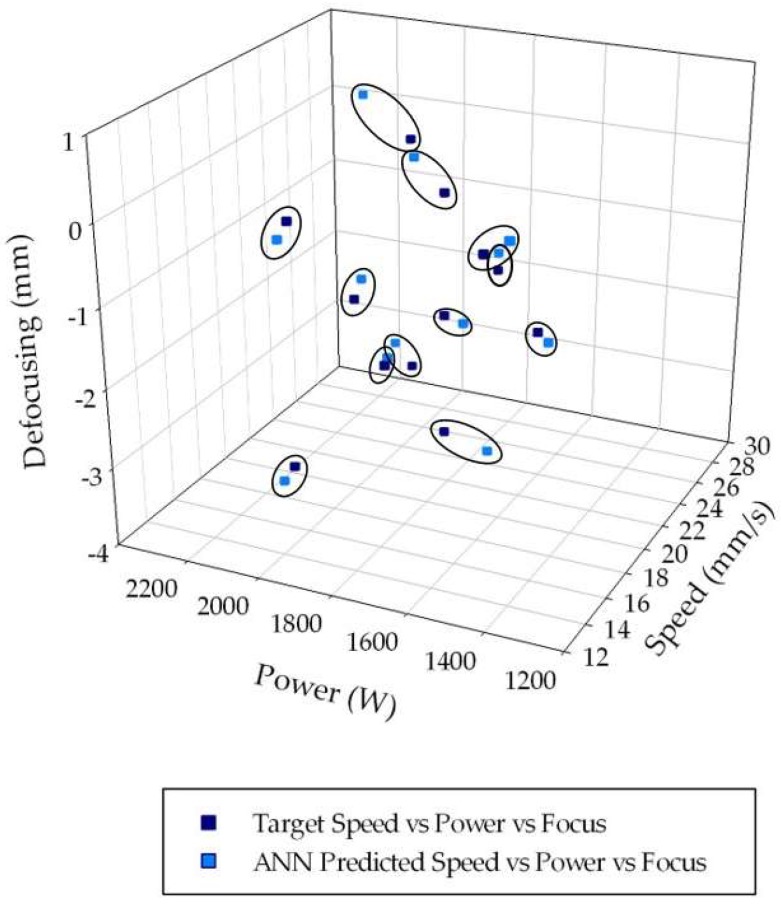
Experimental (blue marks) and artificial neural networks (ANN)-estimated (light blue marks) power values, *p*, for each of the *p* = 12 experimental conditions.

The process parameters predicted by the ANN configuration with 14 hidden layer nodes are reported in Figure 5, together with the actual experimental process parameters. The three-dimensional (3D) graph permits visual examination of the pattern recognition performance of the ANN, which can be considered fully successful. As a matter of fact, it is easier to match each couple of experimental process condition and the corresponding ANN-predicted process condition, which are very closely located within the 3D space. In Figure 5, the matching couples are enclosed in black circles. Concerning the distance between the experimental and predicted values, no preferential direction along any of the three axes was identified, indicating that the small deviations were not due to a more frequently wrong prediction of a specific process parameter (laser power *p*, welding speed *s*, or defocusing distance *f*) compared to the other two parameters.

**Figure 6:**
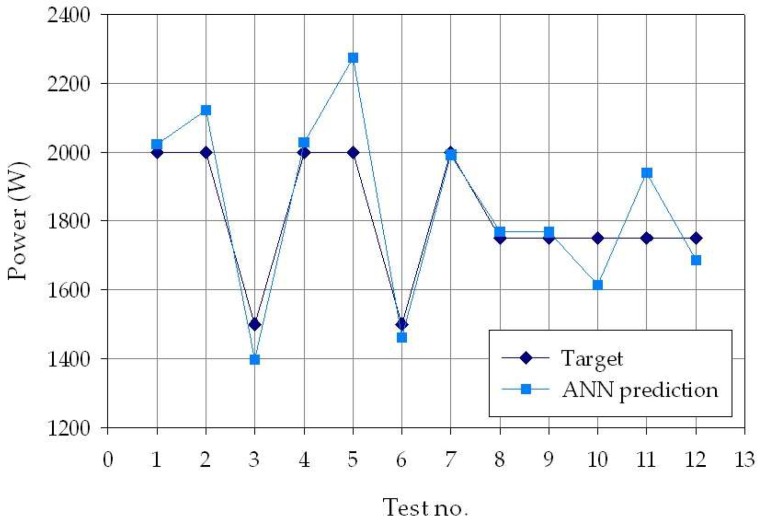
Experimental (blue diamond marks) and ANN-estimated (light blue square marks) laser power values, *p*, for each of the *p* = 30 experimental conditions.

**Figure 7:**
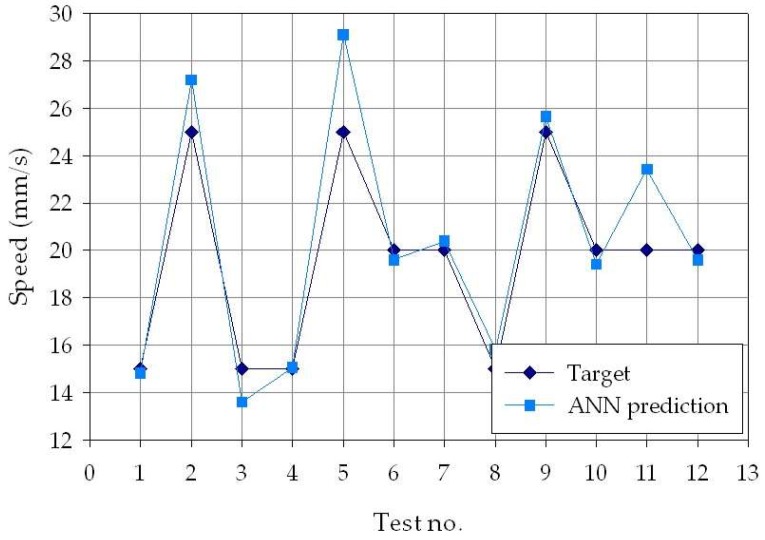
Experimental (blue diamond marks) and ANN-estimated (light blue square marks) welding speed values, *s*, for each of the *p* = 30 experimental conditions.

**Figure 8:**
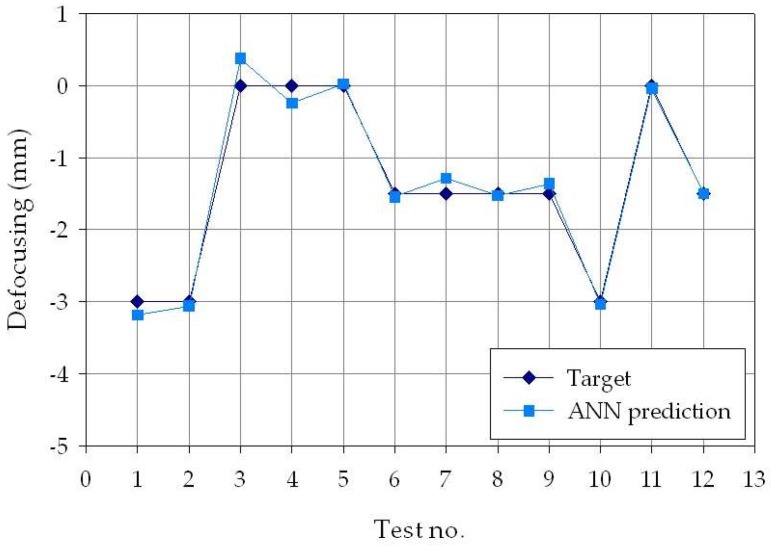
Experimental (blue diamond marks) and ANN-estimated (light blue square marks) defocusing distance values, *f*, for each of the *p* = 30 experimental conditions.

To better visualize the difference between the experimental and ANN-predicted values for each of the three process parameters considered in this study (laser power *p*, welding speed *s*, and defocusing distance *f*), separate charts, i.e., one for each process parameter, are shown in Figure 6, Figure 7 and Figure 8. From the figures, it can be clearly observed that the experimental and the estimated values are close, indicating that the neural network evaluation was accurately performed. These results suggest that the developed cognitive methodology based on ANN can be a valuable tool to support decision making regarding the selection of the suitable laser welding process parameters required to produce weld beads with desired geometry. To further improve the pattern recognition performance of the ANN, additional experimental tests should be performed, with the aim of increasing the number of experimental data to be used for the training phase, as well as widening the range of process parameters taken into consideration.

## 4. Conclusions

In this study, an investigation of the laser welding of Ti–6Al–4V alloy plates was carried out through an experimental testing campaign, under different process conditions, in terms of laser power *p*, welding speed *s*, and defocusing distance *f*, to perform a characterization of the produced weld bead geometry. The final aim was to develop a cognitive methodology able to support decision making about the selection of suitable laser welding process parameters required to produced weld beads with specified geometric features, namely the bead crown width (CW), the root width (RW), the width of the heat-affected zone on the upper surface (HAZup), the width of the heat-affected zone on the lower surface (HAZlow), and the area of the fused zone (FZ). The developed methodology, based on the employment of artificial neural networks, was able to suitably identify correlations between the laser welding process parameters, laser power, welding speed, defocusing distance, and weld bead geometric features, on the basis of the collected experimental data. The aggregated root mean square error values obtained by the best ANN configuration over the *n* = 12 testing cases for the prediction of the laser power, welding speed, and defocusing distance were equal to 117.10 W for laser power, 1.77 mm/s for welding speed, and 0.16 mm for defocusing distance, allowing us to correctly identify, among the experimental testing conditions, the process conditions corresponding to the desired weld bead geometry. As future developments, additional experimental testing campaigns are planned, in order to further enlarge the ANN training set and to identify the optimal process conditions in terms of resulting weld bead geometry.

## Figures and Tables

**Table 1:** Nominal chemical composition of Ti–6Al–4V (wt %).

Al		V		Fe	O_2_	H_2_	C	N_2_	Ti
5.5	6.8	3.5	4.5	0.4	0.2	0.015	0.08	0.05	Balanced

**Table 2:** Main technical features of the laser source.

Laser Source Parameter	Value
Maximum output power (kW)	4.0
Laser light wavelength (nm)	1030
Beam Parameter Product (mm × mrad)	8.0
Focal length (mm)	200
Focus diameter (mm)	300

**Table 3:** Factor levels for the input parameters.

Parameter	Level −1	Level 0	Level 1
*p* (W)	1500	1750	2000
*s* (mm/s)	15	20	25
*f* (mm)	−3	−1.5	0

**Table 4:** Measured values of the weld bead geometric features under the different experimental process conditions (each process condition was tested three times).

Process Conditions			Measured Geometric Features				
*p* (W)	*s* (mm/s)	*f* (mm)	CW (mm)	RW (mm)	HAZup (mm)	HAZlow (mm)	FZ (mm^2^)
2000	15	−3	3.29 3.47 3.61	2.3 2.69 3.02	4.47 4.5 4.49	4.28 4.22 4.04	9.575 9.98 9.25
2000	25	−3	2.72 2.62 2.79	1.67 1.46 1.6	3.45 3.39 3.41	2.39 2.4 2.27	5.291 4.959 4.839
1500	15	0	2.77 2.66 2.9	2.43 2.22 2.37	3.57 3.58 3.51	3.39 3.52 3.33	8.068 8.878 8.168
2000	15	0	3.79 3.95 3.65	3.7 4.08 3.28	4.44 4.76 4.33	4.48 4.83 4.52	11.217 11.928 10.465
2000	25	0	2.82 2.85 2.77	2.42 2.26 2.53	2.34 3.22 3.27	2.98 2.93 3.18	6.847 6.931 7.28
1500	20	−1.5	2.82 2.91 2.92	1.37 1.6 1.78	3.28 3.35 3.38	2.01 2.11 2.49	5.329 5.632 5.983
2000	20	−1.5	3.46 3.57 3.76	2.58 2.78 2.93	3.95 4.07 4.22	3.46 3.55 3.71	8.143 8.135 9.215
1750	15	−1.5	3.79 3.68 3.72	3.08 2.93 2.9	4.47 4.36 4.43	4.05 4.08 4.14	9.259 9.93 10.13
1750	25	−1.5	2.78 2.67 2.79	1.59 1.56 1.28	3.3 3.17 3.29	2.34 2.25 1.97	5.092 4.775 4.721
1750	20	−3	2.88 3.2 3.01	1.18 1.62 1.79	3.48 3.72 3.6	2.05 2.43 2.67	5.844 5.776 6.143
1750	20	0	3.04 2.7 2.65	2.17 2.26 2.22	3.04 3.21 3.15	3.16 3.06 2.87	7.564 7.084 6.586
1750	20	−1.5	2.99 3.17 3.1	2.38 2.36 2.15	3.58 3.7 3.7	3.04 3.12 2.9	7.162 7.572 7.099
